# Genetic variability and evolutionary dynamics of viruses of the family *Closteroviridae*

**DOI:** 10.3389/fmicb.2013.00151

**Published:** 2013-06-26

**Authors:** Luis Rubio, José Guerri, Pedro Moreno

**Affiliations:** Instituto Valenciano de Investigaciones AgrariasMoncada, Valencia, Spain

**Keywords:** *Closterovirus*, *Crinivirus*, *Ampelovirus*, recombination, selection, phylogeny, gene flow

## Abstract

RNA viruses have a great potential for genetic variation, rapid evolution and adaptation. Characterization of the genetic variation of viral populations provides relevant information on the processes involved in virus evolution and epidemiology and it is crucial for designing reliable diagnostic tools and developing efficient and durable disease control strategies. Here we performed an updated analysis of sequences available in Genbank and reviewed present knowledge on the genetic variability and evolutionary processes of viruses of the family *Closteroviridae*. Several factors have shaped the genetic structure and diversity of closteroviruses. (I) A strong negative selection seems to be responsible for the high genetic stability in space and time for some viruses. (2) Long distance migration, probably by human transport of infected propagative plant material, have caused that genetically similar virus isolates are found in distant geographical regions. (3) Recombination between divergent sequence variants have generated new genotypes and plays an important role for the evolution of some viruses of the family *Closteroviridae*. (4) Interaction between virus strains or between different viruses in mixed infections may alter accumulation of certain strains. (5) Host change or virus transmission by insect vectors induced changes in the viral population structure due to positive selection of sequence variants with higher fitness for host-virus or vector-virus interaction (adaptation) or by genetic drift due to random selection of sequence variants during the population bottleneck associated to the transmission process.

## Introduction

There are five basic mechanisms determining the genetic structure and evolution of biological populations: mutation, recombination, natural selection, genetic drift, and migration (Moya et al., 2004). RNA viruses have a great potential for high genetic variability, rapid evolution and adaptation to new conditions and environments due to their rapid replication, generation of very large populations, and high mutation rates (at least 10^5^ times higher than those of their hosts) as a consequence of the lack of proofreading activity of RNA polymerases (Holland et al., 1982; García-Arenal and Fraile, 2011). In many of these viruses, genome recombination and/or reassortment of genomic segments (pseudorecombination) between divergent virus strains or virus species increase genetic variability and accelerate evolution (Chare and Holmes, 2004; Nagy, 2008). The genetic variation generated by mutation and recombination is limited and structured by the other three evolutionary forces: natural selection, genetic drift, and gene flow (Roossinck, 2003; Moya et al., 2004). Natural selection is a directional process by which variants that are fittest in a certain environment will increase their frequency in the population (positive or adaptive selection), whereas variants less fit will decrease their frequency (negative or purifying selection), this process being determined by numerous specific interactions of viruses with their plant hosts (Schneider and Roossinck, 2001), vectors (Power, 2000; Chare and Holmes, 2004) and even with other viruses co-infecting the same plant. Genetic drift consists of stochastic changes in allele frequencies in a finite population due to the random sampling of genes at reproduction (Moya et al., 2004). This supposes a reduction of the genetic variability and fixation of selectively neutral variants, and it has an important effect during severe and rapid reduction in population size produced by population bottlenecks or founder events (Ali and Roossinck, 2008). Genetic drift can occur in different events of the virus life cycle such as virus movement between plant cells (Sacristan et al., 2003; Li and Roossinck, 2004), transmission between plants by vectors (Ali et al., 2006; Betancourt et al., 2008) and interaction between coinfecting viruses (Fraile et al., 1997). Finally, migration (gene flow) among distinct geographical areas, plants or different parts of the same plant is an important factor shaping the genetic structure of viral populations. High migration favors genetic uniformity between populations and thus decreases the global genetic diversity (Moya et al., 2004). While mutation and recombination are intrinsic of the virus genome and its replication and expression systems, natural selection, genetic drift and gene flow are affected by virus biology (e.g., host type and range, means and extent of dispersal), environmental conditions, and population parameters (e.g., population size and history of population bottlenecks).

The study of genetic variability and the evolutionary mechanisms related to the different aspects of the virus biology is crucial to understand virus epidemiology and emergence (Grenfell et al., 2004), designing specific diagnostic tools, and developing efficient and durable strategies of disease control (García-Arenal and McDonald, 2003; Acosta-Leal et al., 2011). Several reviews on evolution of the family *Closteroviridae* have been published, which were focused on macroevolution and taxonomy (Dolja et al., 1994; Agranovsky, 1996; Karasev, 2000; Dolja et al., 2006).

The family *Closteroviridae* is composed of viruses characterized by their long (up to 2000 nm) and flexuous, non-enveloped, polar, virions with two coat proteins, the major (CP), covering most of the genomic RNA, and the minor (CPm) located to one of the virion ends (Agranovsky et al., 1995; Febres et al., 1996; Tian et al., 1999). Its members have the largest genomes of all positive sense RNA plant viruses (up to 20 kb). Although the number and relative position of open reading frames (ORFs) vary between species, there is a common genome organization. ORFs 1a and 1b encode replication-related proteins, with protease, methyl-transferase, helicase, and RNA-dependent RNA polymerase conserved domains. Downstream ORFS include a conserved five-gene module encoding a small hydrophobic protein with affinity to cell membranes, a homolog of the plant heat shock proteins HSP70 (HSP70h), a ~60 kDa protein with a diverged coat protein motif, the CP and the CPm. The functions postulated for the HSP70h are: cell-to-cell movement, involvement in the assembly of multisubunits complexes for genome replication and/or subgenomic RNA synthesis and assembly of virus particles, whereas the ~60 kDa protein is required for incorporation of both HSP70h and CPm to the particle tail (Tian et al., 1999; Satyanarayana et al., 2000; Alzhanova et al., 2001). The genome expression strategy is based on: (I) proteolytic processing of the polyprotein encoded by ORF 1a, (II) +1 ribosomal frameshift for the expression of ORF1b, and (III) expression of the downstream ORFs via the formation of 3′ co-terminal subgenomic RNAs. Presently, there are three genera in the family (Martelli et al., 2011): *Ampelovirus*, *Crinivirus* and *Closterovirus*, although a new genus named *Velarivirus* has been proposed (Al Rwahnih et al., 2012). The characteristics of the accepted genera are:

- *Ampelovirus*. Mealybug-transmitted, monopartite genome, and the CPm gene is located downstream of CP gene or lacking in some species. Viruses studied here are: *Grapevine leafroll-associated virus* 1 (GLRaV-1), GLRaV-3, GLRaV-5, and GLRaV-11 (tentative member), and *Pineapple mealybug wilt-associated virus* 1 (PMWaV-1).
- *Crinivirus*. Whitefly-transmitted, bipartite or tripartite genome, and the CP gene is located upstream of the CPm gene. Viruses analyzed here are: *Blackberry yellow vein-associated virus* (BYVaV), *Cucurbit yellow stunting disorder virus* (CYSDV), *Potato yellow vein virus* (PYVV), *Sweet potato chlorotic stunt virus* (SPCSV), *Tomato chlorosis virus* (ToCV), and *Tomato infectious chlorosis virus* (TICV).
- *Closterovirus*. Mostly aphid-transmitted, monopartite genome, and the CP gene located downstream of the CPm gene. Viruses studied here are: *Citrus tristeza virus* (CTV) and *Grapevine leafroll-associated virus* 2 (GLRaV-2).

In this work we performed an updated analysis of the genetic variation of viruses in the family *Closteroviridae* by analysis of the coat protein genes using nucleotide sequences retrieved from Genbank and present an updated review on the genetic variability and evolutionary processes of the viral populations of members of the family *Closteroviridae*.

## Materials and methods

### Alignement of nucleotide sequences

Nucleotide sequences from worldwide isolates of members of the family *Closteroviridae* were retrieved from GenBank (http://www.ncbi.nlm.nih.gov and http://www.dpvweb.net). The coat protein genes (CP or CPm) were selected because they are present in all viruses and because it is the genomic region for which more sequences are available. Only those viruses with sequences of five or more different isolates were analyzed (Table 1). Multiple alignment was performed with the algorithm CLUSTAL W (Larkin et al., 2007) implemented in the program MEGA 5.05 (Tamura et al., 2011).

**Table 1:** **Genetic diversity and population genetic parameters of viruses of the family *Closteroviridae***.

**Genus**	**Virus**	**Genome^a^**	**NI^b^**	**NC^c^**	**World Areas^d^**							**Population parameters^e^**				**Diversity within genetic groups^f^**					
					***N***	***S***	***A***	***E***	***M***	***F***	***O***	***M***	***D***	***N/S***	***F*_st_**	**I**	**II**	**III**	**IV**	**V**	**VI**
*Ampelovirus*	GLRaV-1	CP	7	5	✓	–	✓	✓	✓	✓	–	0.175	0.093	0.069	N/A^g^	N/A	N/A	0.047	–	–	–
	GLRaV-3	CP	191	9	✓	✓	✓	✓	✓	✓	–	0.256	0.058	0.073	0.092	0.009	0.018	0.037	–	–	–
	GLRaV-4	CP	5	4	✓	–	–	–	✓	✓	–	0.391	0.254	0.096	N/A	0.075	N/A	N/A	–	–	–
	GLRaV-5	CP	79	5	–	✓	–	✓	–	–	–	0.089	0.058	0.171	0.172	–	–	–	–	–	–
	GLRaV-11	CP	15	2	–	✓	–	✓	–	–	–	0.171	0.109	0.127	N/A	0.008	0.006	0.030	N/A	0.073	–
	PMWaV-1	CP	6	5	✓	–	–	–	–	✓	–	0.016	0.008	0.353	N/A	–	–	–	–	–	–
*Crinivirus*	CYSDV	CP	41	13	✓	–	–	✓	✓	✓	–	0.108	0.030	0.008	0.955	0.003	0.002	–	–	–	–
	PYVV	CP	9	2	–	✓	–	–	–	–	–	0.021	0.012	0.214	N/A	–	–	–	–	–	–
	SPCSV	CP	39	8	–	✓	✓	–	–	✓	–	0.379	0.032	0.151	0.327	0.014	N/A	–	–	–	–
	ToCV	CP	23	12	✓	✓	✓	✓	✓	✓	–	0.021	0.011	0.111	0.560	–	–	–	–	–	–
	TICV	CPm	7	5	✓	–	–	✓	–	✓	–	0.007	0.004	0.250	N/A	–	–	–	–	–	–
	BYVaV	CP	34	1	✓	–	–	–	–	–	–	0.058	0.031	0.009	0.225	–	–	–	–	–	–
*Closterovirus*	GLRaV-2	CP	55	7	✓	–	✓	✓	–	✓	–	0.375	0.128	0.161	0.150	0.022	0.030	0.011	0.008	0.009	N/A
	CTV	CP	577	47	✓	✓	✓	✓	✓	✓	✓	0.124	0.073	0.158	0.373	–	–	–	–	–	–

a Genomic region analyzed: CP, coat protein; CPm, minor coat protein.

b NI, number of isolates analyzed for each virus.

c NC, number of countries where the virus isolates were collected.

d World Areas: N, North America; S, South America; A, Sub-Saharian Africa; E, Europe + Mediterranean Africa; M, Middle east; F, Far East; O, Oceania.

e Population parameters: M, maximum nucleotide distance between isolate pairs; D, diversity (mean nucleotide distance between isolate pairs), F_st_ = measure of gene flow.

f Diversity within genetic groups. Groups included isolates that had nucleotide distances higher than 0.1 with respect to all isolates of the other groups.

g N/A, not applicable.

### Analysis of nucleotide sequences

The program MEGA 5.05 was used for: (1) inference of phylogenetic relationships between isolates of each viral species by the neighbor-joining method (Saitou and Nei, 1987), (II) estimation of nucleotide distances between sequence pairs and diversities (mean nucleotide distances) using Kimura-two-parameter as the nucleotide substitution model (Kimura, 1980), and (III) estimation of the ratio between non-synonymous and synonymous substitution (N/S) by the Pamilo-Bianchi-Li method (Pamilo and Bianchi, 1993) to study the role of natural selection at the protein level. N/S ≈ 0 indicates neutral evolution, N/S < 1 negative or purifying selection and N/S > 1 positive or adaptive selection.

The program DnaSP 5.10 (Librado and Rozas, 2009) was used to assess genetic differentiation and the gene flow level between different countries or geographic areas with the statistic *F*_*st*_ (Weir and Cockerham, 1984). *F*_*st*_ can take values from 0, no genetic differentiation and complete gene flow, to 1, complete genetic differentiation as a consequence of null gene flow. Only countries or geographical areas with more than four isolates of the virus analyzed were taken into account.

Recombination between isolates of the same virus was analyzed with the program RDP3 (Martin et al., 2010) that incorporates the recombination-detecting algorithms GENECONV (Padidam et al., 1999), BOOTSCAN (Salminen et al., 1995; Martin et al., 2005), MAXCHI (Smith, 1992; Posada and Crandall, 2001), CHIMAERA (Posada and Crandall, 2001), SISCAN (Gibbs et al., 2000), 3SEQ (Boni et al., 2007), and RDP (Martin and Rybicki, 2000), using their default parameter values. Only those events recognized by at least four different algorithms were accepted as evidence for recombination. The effect of recombination was taken into account during analysis of selection.

## Results

### Genetic variation between virus isolates

**Figure 1:**
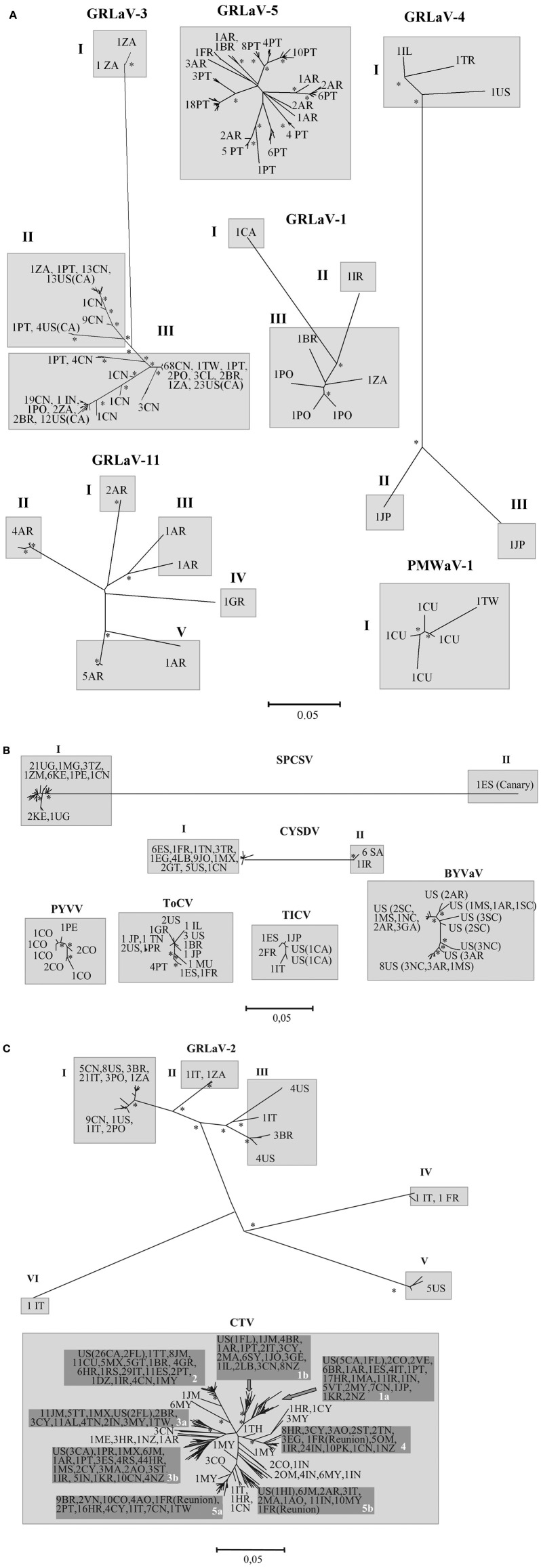
**Neighbor-joining phylogenetic trees of the coat protein genes of different viruses of the family *Closteroviridae*.** Bootstrap values higher than 0.75 are indicated with asterisks. Two-letter codes indicate countries (defined in ISO 3166-1) and/or US states. Numbers preceding the codes indicate the number of isolates analyzed from each country. For each virus, genetic groups are indicated in gray boxes with Roman numerals and include virus isolates having nucleotide distances higher than 0.1 with all isolates from other clades. Subgroups or clades in CTV are indicated in darker boxes with Arabic numbers. **(A)** Genus *Ampelovirus*, **(B)** Genus *Crinivirus*, and **(C)** genus *Closterovirus*.

Figure 1 shows the phylogenetic relationships between isolates of each virus species with branch length indicating genetic distances. For each virus, isolates were classified into genetic groups considered as those clades with all isolates having nucleotide distances higher than 0.1 with respect to all isolates of the other clades. These groups are indicated in gray boxes. Table 1 shows the nucleotide diversities and other population genetic parameters.

In the genus *Ampelovirus*, four out of the six viruses studied comprised three to five genetic groups with distances between isolates of up to 0.391 (Figure 1A and Table 1). GLRaV-1 sequences (Alabi et al., 2011) formed three genetic groups with isolates from: (I) Canada, (II) Iran, and (III) Brazil, Poland and South Africa. GLRaV-3 sequences (Turturo et al., 2005; Fajardo et al., 2007; Chooi et al., 2009; Fuchs et al., 2009; Jooste et al., 2010; Gouveia et al., 2011; Sharma et al., 2011; Wang et al., 2011; Bester et al., 2012; Farooq et al., 2012) also fell into three groups including isolates from: (I) South Africa, (II) California, Portugal, South Africa and China, and (III) California, Brazil, Chile, Portugal, Poland, South Africa, China and Taiwan, with group I diverging much more than groups II and III, and with a maximum nucleotide distance between isolates of 0.256 (Table 1). GLRaV-4, in spite of having CP sequences of only five isolates (Saldarelli et al., 2006) was composed of three genetic groups with isolates from: (I) Israel, Turkey and USA, (II) Japan, and (III) Japan, with group I diverging more than groups II and III and with a maximum nucleotide distance of 0.391. This was the most variable member of the family with a nucleotide diversity value of 0.254 (Table 1). GLRaV-5, with most isolates from Portugal and Argentina, showed moderate genetic variability and all isolates clustered in a single genetic group. GLRaV-11, in spite of having sequences of only seven isolates (six from Argentina and one from Greece), was composed of five genetic groups: four from Argentina and one from Greece, with a nucleotide diversity value of 0.109 (Table 1). Finally, for the pineapple-infecting PMWaV-1, the CP sequences available include four isolates from Cuba and one from Taiwan which were similar and formed a unique genetic group.

The genus *Crinivirus* had the lowest genetic variability of the three genera, with nucleotide diversity values lower than 0.033 (Table 1). Four viruses were composed of isolates clustered in a unique genetic group and two viruses had isolates clustered in two divergent genetic groups (Figure 1B), with isolates within each group having very low variability (diversity below 0.015). The blackberry-infecting BYVaV isolates, all from USA, presented very low genetic variation. The cucurbit-infecting CYSDV was composed of two genetic groups located in: (I) Middle East (Iran and Arabia) and (II) Mediterranean Basin (South Europe, North Africa and Near East), North America and China. Each group had a very low nucleotide diversity (Table 1; Rubio et al., 1999, 2001a; Marco and Aranda, 2005; Sweiss et al., 2007). The potato-infecting PVYV was restricted to South America and presented very low genetic variation. The sweet potato-infecting SPCSV had two very divergent genetic groups with a nucleotide distance of up to 0.379 (Table 1): (I) located predominantly in Eastern Africa, but also with isolates in Peru and China; and (II) located in Western Africa. In our analysis the Western Africa group was represented by only one isolate from the Canary Islands, but analysis of the HSP70h gene of several isolates from Nigeria showed that they were also part of the Western Africa group which diverged from the East Africa group (Fenby et al., 2002). Finally, the tomato-infecting TICV and ToCV showed very low genetic variation in spite of their wide distribution. TICV isolates were from USA, Europe and Japan; and ToCV isolates from Europe, North America, Mediterranean Basin, Africa (Mauritius) and Japan.

In the genus *Closterovirus*, the grape-infecting GLRaV-2 showed high genetic variation (Bertazzon et al., 2010b; Jarugula et al., 2010) like the grape-infecting ampeloviruses, with a maximum nucleotide distance between isolates of 0.375 and a nucleotide diversity of 0.128 (Table 1). GLRaV-2 sequences were classified into six genetic groups including isolates from: (I) China, USA, Brazil, Italy, Poland, and South Africa, (II) Italy and South Africa, (III) USA, Italy, and Brazil, (IV) Italy and France, (V) USA, and (VI) Italy (Figure 1C). Finally, the citrus-infecting CTV, the best studied virus in the family, with CP sequences of almost 600 worldwide isolates (Albiach-Martí et al., 2000; Rubio et al., 2001b; Alavi et al., 2005; Roy et al., 2005; Sorrentino et al., 2005; Papayiannis et al., 2007; Iglesias et al., 2008; Jiang et al., 2008; Černi et al., 2009; Herrera-Isidrón et al., 2009; Oliveros-Garay et al., 2009; Fisher et al., 2010; Harper et al., 2010; Matos et al., 2013), showed a moderate genetic variability. All isolates fell into a single genetic group, albeit this could be divided into five to seven subgroups (Figure 1C), some of them very homogeneous (isolates with almost identical sequences). There was no association between the subgroups and the geographic origin of CTV isolates. In our analysis, all biologically characterized isolates in the subgroup 1a were severe and induced stem pitting in sweet orange and/or grapefruit, whereas those characterized in the subgroup two were mild, inducing only weak symptoms in Mexican lime. Other subgroups included mild and severe isolates with no association between symptoms and genetic distance between isolates being observed. For example, about 43, 70, 40, 25, and 19% of the biologically characterized isolates in subgroups 1b, 3b, 3a, 4, and 5a, respectively, were mild, and 57, 30, 60, 75, and 81%, respectively, were severe and incited stem pitting in grapefruit or sweet orange (Alavi et al., 2005; Papayiannis et al., 2007; Černi et al., 2008; Harper et al., 2009; Nolasco et al., 2009; Biswas et al., 2012; Hančević et al., 2013).

### Recombination

Most of the analyzed viruses showed no recombination in the CP (Table 2). GLRaV-3 had 14 isolates out of 191 with a recombinant CP which resulted from three different recombination events involving different parental sequences and recombination sites (approximate nucleotide positions 300, 450, and 500 of the CP gene). All recombinations involved isolates from China, except one that had a parental sequence from Chile. GLRaV-11 had four isolates out 15 with the same recombination at nucleotide position 500 involving the same parental sequences. CTV comprised 63 out of 577 isolates with a recombinant CP resulting from two recombination events with respect to the parental sequences and recombination sites (Table 2). CTV isolates from South America, North America, Africa, and Asia showed the same recombination indicating that this corresponds to an ancient event that occurred before the recombinants spread worldwide.

**Table 2:** **Recombination events detected in the coat protein genes of viruses of the family *Closteroviridae***.

**Genus**	**Virus**	**Genome^a^**	**NI^b^**	**NR^c^**	**RE^d^**	**Sites^e^**
*Ampelovirus*	GLRaV-1	CP	7	0	0	–
	GLRaV-3	CP	191	14	3	X-500, 450-X, 300-X
	GLRaV-4	CP	5	1	1	500-X
	GLRaV-5	CP	79	0	0	–
	GLRaV-11	CP	15	4	1	400–500
	PMWaV-1	CP	6	0		–
*Crinivirus*	CYSDV	CP	41	0	0	–
	PYVV	CP	9	0	0	–
	SPCSV	CP	39	0	0	–
	ToCV	CP	23	0	0	–
	TICV	CPm	7	0	0	–
	BYVaV	CP	34	0	0	–
*Closterovirus*	GLRaV-2	CP	55	0	0	–
	CTV	CP	577	63	2	200-X, 400-X

a Genomic region analyzed: CP, coat protein; CPm, minor coat protein.

b NI, number of isolates analyzed for each virus.

c NR, number of recombinant isolates.

d Number of different recombination events.

e Approximate recombination sites. X means an unknown recombination site outside of the coat protein gene.

### Natural selection

The ratio of non-synonymous to synonymous substitutions (N/S values in Table 1), were low indicating functional or structural constraints for amino acid changes. The selection pressure was particularly strong for CYSDV, with N/S = 0.008 and composed of two genetic groups separated by a genetic distance of about 0.1. Indeed only 0.2% of the nucleotide changes between the two groups produced amino acid changes. In some cases the number of synonymous substitutions was very low (not shown) suggesting also constraints for nucleotide changes that could affect thermodynamic stability of RNA, codon usage bias for translation efficiency, secondary structure, activation of gene silencing, or RNA-RNA or RNA-protein interactions (Cuevas et al., 2012).

### Migration (gene flow)

The presence in one geographic region of a diverse virus population with some isolates similar to those of other regions is usually indicative of a possible dispersion center for that virus (Bateson et al., 2002). Our analyses showed that GLRaV-3 had isolates from South Africa in the three genetic groups making plausible to consider South Africa or nearby areas as a dispersion center of this virus. However, this could be a secondary dispersion center since South Africa is far from the host (genus *Vitis*) origin region and likely initial dispersion area. GLRaV-11 had four genetic groups with isolates from Argentina and one group with one isolate from Greece, suggesting that Argentina could be a dispersion center for this virus. Finally, GLRaV-2 had six genetic groups with isolates from Italy distributed in five groups and isolates from USA in three groups which pointed at both countries as possible dispersion zones. Another explanation is that the locations with divergent isolates received them via importation of infected grapevine material from different areas. In most cases, the phylogenetic relationships among viral isolates were not geographically structured and often isolates from distant regions were genetically very close (Figure 1), indicating long distance movement probably by international traffic of virus-infected plant material (Rubio et al., 2001b; Angelini et al., 2004; Alabi et al., 2011).

To estimate the degree of virus migration or gene flow, the statistic *F*_*st*_ was calculated (see above). The global gene flow was high for GLRaV-3, moderate for GRLaV-5 and GRLaV-2, and low for criniviruses and CTV (Table 1). A more detailed analysis was performed by comparing virus subpopulations of different areas two by two (not shown). GRLaV-1 subpopulations from California, Washington, and New York (Alabi et al., 2011) had an infrequent genetic exchange. CYSDV has a null gene flow between the Middle East and the rest of the world, but the gene flow was very high between Spain, Near East, and North America (*F*_*st*_ = 0.000), albeit this could be due to a unique migration event given the genetic stability of this virus. SPCSV showed also a high geographical structure between the two genetic groups, but availability of only one isolate in one group precluded gene flow analysis between both groups. In group I, the comparison of three neighboring countries of East Africa showed a high gene flow between Uganda and Kenya (*F*_*st*_ = 0.027) but very low between them and Tanzania (*F*_*st*_ ≈ 0.450). In spite of its low genetic diversity, ToCV showed a low gene flow between Europe and North America (*F*_*st*_ = 0.560). GRLaV-2 with a moderate global flow showed a puzzling migration pattern which did not correspond to geographic proximity. Thus, extensive gene flow occurred between Poland and China and between Italy, Brazil and USA but this was very low between Italy and Poland. Finally, CTV, the best documented virus with isolates from 47 countries showed different degrees of gene flow which were not correlated with geographic distance. Some subpopulations had a high gene flow (*F*_*st*_ < 0.100), e.g., California, Mexico, Spain, Italy, and Portugal, or Brazil, Angola, China, and Portugal, whereas other subpopulations were almost isolated (*F*_*st*_ > 0.300) such as Cuba (although with a moderate gene flow, *F*_*st*_ ≈ 0.150, with Mexico and Guatemala), Trinidad and Tobago, and Argentina. Several reports showed that genetically and biologically divergent isolates of CTV have been introduced one or several times in Iran, Sicily (Italy), Cyprus, and Dominican Republic (Alavi et al., 2005; Davino et al., 2005; Papayiannis et al., 2007; Matos et al., 2013).

## Discussion

The genetic variability for each virus analyzed was different, although it must be taken into account that in some cases the number of sequences available was low and/or these were from a few geographical locations. The most variable viruses were the grapevine-infecting ampeloviruses GRLaV-1, 4, and 11 and the closterovirus GLRLaV-2, whereas criniviruses showed very low genetic variation. In general, the viruses of the family *Closteroviridae* showed a low genetic diversity or were comprised of genetic groups with very low within-group nucleotide diversity, as found in many other plant viruses (García-Arenal et al., 2001), due to strong negative selection. The existence of a few genetically homogeneous genetic groups suggests that the sequence space of these viruses may be restricted to a few narrow peaks in the adaptive or fitness landscape (Wright, 1932). A high level of covariation at molecular level (the coordinated change of certain nucleotides in response to the change of other nucleotides to maintain biologically relevant structures and functions) could explain this discontinuous adaptive landscape (Gultyaev et al., 2000). Reduced fitness of chimeras between CTV strains from different genetic subgroups occupying distinct adaptive peaks (Satyanarayana et al., 1999) support this notion. The temporal genetic stability reported for CTV (Albiach-Martí et al., 2000; Rubio et al., 2001b; Silva et al., 2012) and CYSDV (Marco and Aranda, 2005; Rubio et al., 2001a) supports the existence of a strong negative selection. There have been many studies trying to associate genetic relationships and the pathogenicity characteristics of CTV variants (Sambade et al., 2003; Hilf et al., 2005; Nolasco et al., 2009). Although sequence of the CP gene does not appear associated with pathogenicity characteristics in many subgroups, separation between mild and severe stem pitting isolates found in other genomic regions and analysis of phylogenetic networks suggest that pathogenicity has been an important evolutionary force in CTV populations (Martin et al., 2009).

Viruses of the family of *Closteroviridae* are transmitted by insect vectors which favor mixed infections between different viruses or strains of the same virus. Mixed infections may have important evolutionary implications since they can affect the within-isolate population of virus variants (quasispecies) and allow interaction and/or recombination between different virus entities which can affect to pathogenicity and adaptability.

Recombination has played an important role in the evolutionary history of the family *Closteroviridae*. It has been postulated that the ancestor of this family was a smaller filamentous virus composed of three genes encoding replication-associated proteins, the p6-like movement protein and a single coat protein (Dolja et al., 2006). During evolution new genes were incorporated to the genome by two means: (i) recombination with cellular mRNAs, e.g., HSP70h, or with RNAs of other viruses, e.g., the leader proteinase (L-Pro) from potyviruses, and (ii) unequal recombination between two genomic RNA copies (or involving subgenomic RNAs) of the same virus which produced gene duplication, e.g., the genes encoding the ~60 kD and CPm proteins evolved after being generated as duplicates of the CP gene. Recent cases of recombination-mediated gene gain have occurred in the criniviruses SPCSV and *Beet pseudoyellows virus* (BPYV), both with isolates differing in the number of genes (Tzanetakis and Martin, 2004; Cuellar et al., 2008). Unequal recombination also generated the multipartite genome of criniviruses.

Our analyses indicated homologous recombination in the coat protein gene between divergent genetic variants of GLRaV-3, GLRVaV-11, and CTV. It is clear that more recombination events would be found if additional genomic regions were analyzed. Homologous recombination between divergent isolates of the same virus has been reported for CTV (Rubio et al., 2001b; Sambade et al., 2003; Hilf, 2005; Roy et al., 2005; Vives et al., 2005; Weng et al., 2007; Gomes et al., 2008; Martin et al., 2009; Roy and Brlansky, 2009, 2010; Harper et al., 2010; Melzer et al., 2010; Scott et al., 2013), GLRaV-2 (Alabi et al., 2011) and *Raspberry leaf mottle virus* (McGavin and MacFarlane, 2010); the crinivirus BYVaV (Poudel et al., 2012) and the ampeloviruses GLRaV-3 (Turturo et al., 2005), GLRaV-4 (Thompson et al., 2012) and GLRaV-5 (Turturo et al., 2005; Farooq et al., 2012). Moreover, phylogenetic network analysis showed that homologous recombination must be an important evolutionary force for CTV (Martin et al., 2009). Population analyses showed CTV isolates containing a heterogeneous population of diverged virus strains and recombinants at low frequency (Vives et al., 2005; Weng et al., 2007; Scott et al., 2013), suggesting that these recombinants did not have a selective advantage (more fitness) with respect to the parental sequences. In some cases artificial chimeras of two genetically and biologically divergent CTV isolates failed to infect citrus (Satyanarayana et al., 1999) suggesting that only some recombinants are viable. Also, homologous recombination seems to have occurred between CTV and another unknown closterovirus given the unusual disparity in the divergence of CTV isolates between the two halves of CTV genome: ~0.1 for the 3'-moiety and ~0.3 for the 5'-moiety (Mawassi et al., 1996; Vives et al., 1999).

Unequal recombination seems to occur frequently during replication as evidenced by the large number of defective RNAs (D RNAs) detected in the closterovirus CTV (Mawassi et al., 1995; Ayllón et al., 1999a; Mawassi et al., 2000; Che et al., 2002, 2003), the criniviruses *Lettuce infectious yellows virus* (LIYV) (Rubio et al., 2000b, 2002), PYVV (Livieratos et al., 2004; Eliasco et al., 2006) and SPCSV (Kreuze et al., 2002; Cuellar et al., 2008), and the ampelovirus GLRaV-3 (Ling et al., 1998). D RNAs are deletion forms of virus genomic RNAs that retain the replication signals but require the parental virus for replication. The existence of direct repeats, secondary structure or AT-rich regions at the junction site of some D RNAs suggested template-switching as a plausible mechanism for recombination (Ayllón et al., 1999a; Rubio et al., 2000b). In this model the D RNAs are generated by a translocation event in which the polymerase, together with the nascent RNA strand, falls off the template strand probably at regions of secondary structure and RNA synthesis reinitiates at a different site with identical or similar nucleotide sequence to the jumping site. Also a weak base pairing in A/U-rich regions can facilitate the release and/or the re-annealing of the incomplete nascent RNA by formation of a temporary bubble (Nagy and Bujarski, 1997). The junction site of some CTV D RNAs coincided with the transcription start site of subgenomic RNAs (Yang et al., 1997) suggesting their involvement in recombination and in the genome modular evolution of the family *Closteroviridae* (Bar-Joseph et al., 1997). The stochastic nature of D RNA generation as replication errors was evidenced by the great variety of D RNAs generated after protoplast inoculation with RNA transcripts of the two LIYV genomic segments (Rubio et al., 2002).

The genetic variation and structure of viruses within an infected plant (considered as a virus isolate) also provides important information to understand viral evolution. This has been studied for GRLaV-1 (Alabi et al., 2011), GRLaV-3 (Turturo et al., 2005; Esteves et al., 2012), CYSDV (Rubio et al., 1999, 2001a), ToCV (Lozano et al., 2009), GRLaV-2 (Bertazzon et al., 2010b; Jarugula et al., 2010), and CTV (Ayllón et al., 1999b, 2006; d'Urso et al., 2000; Kong et al., 2000; Rubio et al., 2001b; Davino et al., 2005; Hilf et al., 2005; Melzer et al., 2005; Silva et al., 2007; Gomes et al., 2008; Iglesias et al., 2008; Černi et al., 2009; Oliveros-Garay et al., 2009; Matos et al., 2013; Wu et al., 2013). Analysis of nucleotide sequences or molecular markers such as single-strand conformation polymorphism (Rubio et al., 1996) of a certain number of clones obtained from RT-PCR products showed that most viral isolates had populations composed of a predominant sequence variant and different one- or two-nucleotide mutants in a very low frequency. These mutant clouds are predicted by the quasispecies model as a consequence of the high error frequency in RNA replication and have been described for some animal and plant viruses (Domingo and Holland, 1997). However, some viral isolates had two or more divergent variants, some of which were genetically similar to variants predominant in other viral isolates, suggesting mixed infection by two different strains. These strain mixes that have been found in the closteroviruses CTV (Kong et al., 2000; Rubio et al., 2001b; Sambade et al., 2002; Ayllón et al., 2006; Iglesias et al., 2008) and GLRaV-2 (Bertazzon et al., 2010a), and in the ampeloviruses GLRaV-3 (Farooq et al., 2012) and GLRaV-5 (Esteves et al., 2012), must be common in vector-transmitted viruses infecting long-lived woody hosts. Infection of the same cell with diverged virus strains is a requisite for detectable recombination events to occur.

Co-infection of two viruses or virus strains in mixed infections may have additional evolutionary consequences resulting from their interactions. Sometimes interaction is synergistic, inciting more severe symptoms and increased fitness (virus accumulation) of one or both variants in comparison with single infections. This effect seems to be due to the supression of a host defense mechanism, e.g., gene silencing, by one of the viruses that inhibits accumulation of the other virus in single infections (Palukaitis, 2011). Several cases of synergism have been described between criniviruses and other plant viruses. BYVaV increases concentration of Blackberry virus Y (BVY, genus *Brambyvirus*, family *Potyviridae*) in mixed infections (Susaimuthu et al., 2008). CYSDV enhanced multiplication of *Cucumber vein yellowing virus* (CVYV, genus *Ipomovirus*, family *Potyviridae*) and increased symptom severity in mixed infections (Gil-Salas et al., 2012). In sweet potato, SPCSV increased multiplication of several viruses of the genera *Potyvirus* (*Sweet potato feathery mottle virus*, *Sweet potato latent virus* and *Sweet potato mild speckling virus*), *Ipomovirus* (*Sweet potato mild mottle virus*), *Cucumovirus* (*Cucumber mosaic virus*), and putative members of the genus *Carlavirus* (*Sweet potato chlorotic fleck virus* and C-6 virus) in double and triple infections, which was associated to an increase in the severity of symptoms. In some cases SPCSV titer was reduced indicating an antagonistic interaction (Karyeija et al., 2000; Mukasa et al., 2006; Untiveros et al., 2007). Co-infection of ToCV and *Tomato spotted wilt virus* (TSWV, genus *Tospovirus*) in tomato plants susceptible to both viruses resulted in rapid death of the plants, with a pronounced enhancement of ToCV accumulation, whereas TSWV accumulation was not altered. However, in tomato cultivars carrying the *Sw-5* gene that confers resistance to TSWV, preinfection with ToCV resulted in TSWV resistance breakage, a phenomenon not observed when plants were simultaneously co-inoculated with both viruses. This suggested that a threshold level or a time lapse is needed for ToCV to interfere or downregulate the defense response in the TSWV-resistant plants (García-Cano et al., 2006). Finally, co-infection of the two tomato-infecting criniviruses TICV and ToCV altered accumulation of each virus in a host-specific manner. While in *Nicotiana benthamiana* the TICV titer increased and the ToCV titer decreased, in *Physalis wrightii* the titers of both TICV and ToCV decreased in comparison with the corresponding single infections (Wintermantel et al., 2008). In summary, sinergistic co-infections lead to higher accumulation of at least one of the viruses and may acelerate its adaptation to an initially difficult host.

Other times antagonist interactions may produce a fitness decrease of one or the two viruses. In some cases, previous infection by one viral isolate prevents or hamper subsequent infection by other viral isolate (superinfection exclusion). This phenomenon has been exploited as a disease control strategy named cross protection consisting of preinoculation of the plant with a mild isolate to protect it against damage caused by infection with a virulent isolate. This interaction is most common between genetically related viruses and it has been hypothesized that it might be caused by competition for host resources or because previous infection would trigger the gene silencing antiviral defence of the plant that would impair infection by the second virus (Palukaitis, 2011). CTV cross protection has been efficient for disease control only in some areas (e.g., South Africa and South America) and with some citrus varieties, whereas it has shown limited success in other areas or with other varieties, indicating that (i) cross protection probably depends on the varieties, CTV strains and environmental conditions prevalent in each region (Moreno et al., 2008), and (ii) it is unlikely that CTV cross protection is ruled only by the gene silencing reaction triggered by the pre-inoculated isolate. Indeed preinoculation of citrus plants with artificial hybrid CTV virions containing some genomic segments of the challenging isolate failed to exclude superinfection by this isolate, with only isolates of the same strain being excluded (Folimonova et al., 2010). Demostration that the CTV p33 protein is necessary for superinfection exclusion (Folimonova, 2012) further supports the hypothesis that antiviral silencing reaction triggered by mild isolate pre-inoculation may not be the main mechanism for CTV cross protection. Finally, co-inoculation with a mild and a severe isolate genetically divergent usually resulted in severe symptom expression and predominance of the severe isolate, indicating cross protection failure between divergent isolates and higher fitness of the severe isolate (Roistacher and Dodds, 1993; Sambade et al., 2007; Velazquez-Monreal et al., 2009). Therefore, whatever the cross protection mechanism will be, massive use of cross protecting CTV isolates in some citrus areas is doubtless an important determinant of the viral population structure and an evolutionary factor.

Interactions with the host are one of the most important factors in virus evolution. The tandem of leader proteases of GLRaV-2 seems to have evolved to facilitate infection of this virus in grapevine, a woody perennial host (Liu et al., 2009). Another host effect is the CTV codon usage adaptation to citrus that has also been found in other closteroviruses infecting woody plants but not in those infecting herbaceous hosts (Cheng et al., 2012). Biological and genetic variations of some CTV isolates have been also observed after host change (Ayllón et al., 2006; Scott et al., 2013). Thus, graft-transmission of the mild isolate T317 from citron to sweet orange originated the virulent isolate T318. When T318 was transmitted back to citron, it remained virulent and had the same population structure as it had in sweet orange (Rubio et al., 2000a). This suggests that a minor severe variant contained in the mild isolate T317 was established in sweet orange and became predominant in the T318 population by genetic drift during a transmission bottleneck. This severe variant was as fit in citron as the mild variant (well-adapted to citron and sweet orange). However, when 3 years latter the mild isolate T317 was transmitted again to sweet orange, the new isolate T317D was also mild, albeit the population structure had changed as detected by SSCP analysis. When T317D was transmitted back to citron, its population structure changed back to become indistinguishable from that of the original isolate T317 (Rubio et al., 2000a). This suggests that the severe variant was not sorted in this occasion but some of the mild variants were positively selected as an adaptation to sweet orange. Predominance of these mutants was reverted after back transmission to citron probably as a result of fitness trade-off by host specialization (Woolhouse et al., 2001).

Finally, interactions with the vector can also have an important effect in viral evolution. The association between phylogenetic relationship among members of the three genera of the *Closteroviridae* family and their type of insect vector (mealybugs for the genus *Ampelovirus*, whiteflies for *Crinivirus* and aphids for *Closterovirus*) likely reflects vector adaptation as a driving force in the diversification in this family (Karasev, 2000). In criniviruses, an emergent group of viruses whose expansion has been linked to the rapid spread of their whitefly vectors (Wisler et al., 1998), the specificity of the association between virus species and whitefly species is a main factor determining the geographical distribution of the different criniviruses. Thus, the displacement of the crinivirus BPYV by CYSDV in Spain has been associated with the increasing populations of *Bemisia tabaci* in comparison with *Trialeurodes vaporariorum* (Berdiales et al., 1999). Also disappearance of LIYV in Southern California has been associated with the displacement of the biotype A of *B. tabaci* by the biotype B, a very poor vector of LIYV (Cohen et al., 1992; McLain et al., 1998; Wisler et al., 1998). Geographical distribution of TICV and ToCV also seems to depend on distribution of the whitefly vectors (Wintermantel, 2008). Interestingly, mixed infections of ToCV and TICV allowed transmission of TICV by the non-vector *T. abutilonea* (Wintermantel, 2008), indicating an interaction of these viruses during transmission. In ampeloviruses, there is no evidence of vector–virus specificity in the mealybug transmission of different grapevine viruses (Tsai et al., 2010). In closteroviruses, changes in aphid transmissibility of the local CTV isolates have been observed along the years. In the early 1950s, the transmission rate of CTV by *Aphis gossypii* Glover was very low but this increased in 1960–1970 in Israel and California. (Bar-Joseph and Loebenstein, 1973; Roistacher et al., 1980), suggesting an adaptation and coevolution of CTV to this vector. Brazil and Dominican Republic severe CTV variants are preferentially transmitted by *Toxoptera citricida* in comparison with the mild components (Brlansky et al., 2011). The absence of this vector in some geographical areas could explain in part why severe CTV isolates are less common in these areas, a situation that may change after introduction of the brown citrus aphid as observed in several countries (Garnsey et al., 2000; Powell et al., 2003). Differences in transmissibility of CTV isolates by different aphid species (Raccah et al., 1976, 1980; Hermoso de Mendoza et al., 1988; Broadbent et al., 1996) indicate the vector exerts a selective pressure and it is an important factor shaping CTV populations. There are evidences that aphid transmission can induce changes in the population structure and/or biological characteristics of individual CTV isolates (Kano and Koizumi, 1991; Ayllón et al., 1999b, 2006; d'Urso et al., 2000; Van Vuuren et al., 2000; Sentandreu et al., 2006; Sambade et al., 2007; Roy and Brlansky, 2009; Velazquez-Monreal et al., 2009; Ananthakrishnan et al., 2010). Comparison of genetic diversity in plants and in aphids showed the occurrence of an important bottleneck for the CTV population during aphid transmission (Nolasco et al., 2008). All these observations indicate that vectors have an important effect in the evolution of closteroviruses by the interplay of natural selection imposed by vector-virus interactions and genetic drift by population bottlenecks during transmission between plants. Also, the movility and dispersibility of vectors determine the level of gene flow between close geographical areas. Thus, the rapid spread of some white-flies which could account in part for the low genetic variation and wide distribution areas of the criniviruses transmitted by them.

## Conclusions

The increased number of nucleotide sequences and the availability of more sophisticated analytical tools allows a better understanding of the evolution, population genetics and epidemiology of the viruses in the family *Closteroviridae*. Analysis of the genetic variability and population structure shows a limited genetic variation as in other plant viruses. This seems to occur mainly by a strong negative selection, indicated by the low number of non-synonymous substutions with respect to the synonymous substitutions in the three genera of this family. Some viruses, had isolate groups with a great genetic divergence between groups but each group being very homogeneous. This suggests that their sequence space is restricted to a few sharp adaptive peaks and that covariation between different nucleotide positions occurs, as suggested by decreased fitness of chimeras of different CTV strains. Long distance movement or gene flow may have contributed in some cases to this low genetic variation.

Although most viral isolates were composed of one major variant and a population of genetically related variants forming a quasispecies structure, some isolates had divergent variants originated from mixed infections of different strains which can affect symptom expression. Interaction between different viruses or variants from the same virus has been found in some cases. The antagonism between close variants of the same virus is the base for cross protection to control damage from severe CTV strains (Moreno et al., 2008). Another element in the virus evolution is the interaction between different virus species, as the synergistic or antagonist interactions observed between the two tomato infecting criniviruses (ToCV and TICV) in different hosts (Wintermantel et al., 2008). Also, viruses of the genus *Crinivirus* produce increased symptom expression and accumulation of viruses from other families in mixed infections (Karyeija et al., 2000; Mukasa et al., 2006; Untiveros et al., 2007).

Changes in the population structure caused by vector transmission or host change, either by selection of viruses or genetic variants of the same virus or by genetic drift, could explain the appearance of virulent CTV isolates, or the emergence of some viruses in new areas, e.g., criniviruses, associated to their whitefly vector spread.

Recombination between divergent genotypes have been described in the genera *Closterovirus* and *Ampelovirus*, which have played an important role in their evolution by increasing genetic diversity and adaptability.

### Conflict of interest statement

The authors declare that the research was conducted in the absence of any commercial or financial relationships that could be construed as a potential conflict of interest.
